# Effectiveness of Diabetes Case Conferencing Program on Diabetes Management

**DOI:** 10.5334/ijic.6545

**Published:** 2023-01-25

**Authors:** Reetu Zarora, David Simmons

**Affiliations:** 1 School of Medicine, Western Sydney University, Diabetes Obesity and Metabolism Translational Research Unit, Macarthur Clinical School, Campbelltown, New South Wales, Australia; 2 School of Medicine, Western Sydney University, Diabetes Obesity and Metabolism Translational Research Unit, The Translational Health Research Institute, Macarthur Clinical School, Campbelltown, New South Wales, Australia

**Keywords:** diabetes mellitus, Type 2 diabetes mellitus, clinical effectiveness, endocrinologist, integrated health care system, multidisciplinary care team, primary health care

## Abstract

**Aims:**

Diabetes case conferencing is where an endocrinologist visits a general practitioner (GP) to advise on the care of patients with diabetes. Past case conferencing studies have reported improved diabetes management and clinical outcomes in primary care. This study investigated the effectiveness of a diabetes case conferencing program in South Western Sydney, Australia.

**Methods:**

Complex diabetes cases were referred by general practitioners to a visiting endocrinologist for review after obtaining patient consent. The patient was not usually present. After the case discussion, a diabetes management plan was developed jointly by the general practice/specialist team. Clinical data were compared at baseline and each year up to three years (2017–2020) after the consultation using paired t-test. The primary outcome was HbA1c.

**Results:**

Clinical data were collected for 645/775 patients (mean age 64 ± 15(SD) years; 351 (54.4%) males from 40/43 general practices; 96.4% had type 2 diabetes; 6.5% were insulin treated, 54.3% non-insulin treated, 31.5% both insulin and non-insulin treated and 3.4% diet only. There were reductions in HbA1c by 1.0 ± 1.7% (11 ± 19 mmol/mol) (p < 0.001), systolic blood pressure 8.2 ± 18.1 mmHg (p < 0.001), diastolic blood pressure 2.7 ± 11.6 mmHg (p < 0.001), total cholesterol 0.2 ± 1.7 mmol/l (p = 0.007), low-density lipoprotein 0.2 ± 1.0 mmol/l (p < 0.001), weight 3.3 ± 10.1 kg (p < 0.001) and body mass index (BMI) 1.3 ± 3.5 kg/m^2^ (p < 0.001).

**Conclusions:**

Glycaemia, weight and cardiovascular risk factors improved following case conferencing consultations in a primary care setting.

## Introduction

The diabetes epidemic is a growing health challenge in both Australia and globally. It is a chronic and complex disease and was among the top 10 causes of death at a global level in 2019 [1]. Type 2 diabetes accounts for 90% of all diabetes cases [2]. Diabetes can lead to premature mortality and long-term microvascular and macrovascular complications particularly if undertreated. In 2019, at least USD 760 billion dollars were spent in diabetes related health expenditure globally, posing a public health systems challenge [2].

Mechanisms to improve integration between primary and secondary care are particularly important when managing complex cases in countries where the diabetes care is fragmented [3]. Specialist outreach clinics in general practices increase accessibility and improve health outcomes particularly in rural areas with limited access to specialist and hospital services [4]. Primary care (general practice or family physician practice) is at the centre of the healthcare system in many countries, requiring integration with secondary and tertiary care to close the gap for better patient health outcomes [5].

Case conferencing/multidisciplinary case conference is where patient histories/cases are reviewed (including e.g. medications, recent blood tests, diabetes duration, diabetes complications), their multidisciplinary care needs are identified, and the best pathway forward is planned [6]. Several studies have evaluated diabetes management provided by a multidisciplinary team with the patient present during the consultation. Both virtual/videoconferencing and face-to-face consultations have reported improved glycaemic outcomes [7,8,9,10,11,12,13]. This is the first study to evaluate a diabetes case conferencing program without patients’ presence/participation in a diabetes multidisciplinary consultation.

In Australia, approximately 1.2 million had diabetes in 2017–18 with the prevalence higher among those aged 65–74 years (15.4%) and in the lowest socioeconomic areas (7.0%) [14]. Diabetes contributed to 11% of deaths in Australia and an estimated 1.2 million hospitalisations in 2017–18 (principal and/or additional diagnosis) [15]. Type 2 diabetes is commonly diagnosed and managed in a primary care setting. In Australia, funding is available to provide joint specialist consultations to manage complex and chronic cases [6] of which diabetes is one example. The role of GPs is crucial in diabetes management and a proactive review of patient care with a specialist has improved diabetes management in primary care in a number of local initiatives [10,11,12].

Diabetes is a significant health burden in South Western Sydney, a multi-ethnic area with 966,450 people (in 2016), 10.8% of whom have diabetes, and with increasing prevalence estimates over time [16]. The aim of this diabetes case conferencing program is to enhance diabetes management in primary care, provide more comprehensive care for patients with diabetes, avoid hospital admissions and to upskill the GPs without the patient being physically present. Chronic Disease Management- multidisciplinary case conferencing is an established service by the Australian Government, Department of Health [6] where Medicare rebates are provided for the services provided by GPs and other healthcare professionals to organise, coordinate, and participate. This study aims to evaluate the clinical effectiveness of diabetes case conferencing program across South Western Sydney.

## Research methods

### Study design and setting

Participating sites were general practices across seven Local Government Areas (LGA) in South Western Sydney who referred patients for a case conferencing consultation. All general practices (n = 422) were invited to participate through the usual communication channels of the Primary Health Network. Baseline clinical data were collected from the participating general practices at the time of consultation and the follow-up clinical data were collected after at least 6 months of the initial consultation. Between 2017–2019, case conferencing endocrinologists were assigned a LGA to provide consultations. There were up to three endocrinologists at any time sharing a 0.7 full time equivalent workload. Endocrinologists visited the general practices for a face to face case conferencing session without an extra cost to the patient. A third healthcare professional from the hospital (diabetes educator) or general practice also participated in the case conferencing, who could be a dietitian, diabetes educator, podiatrist or a practice nurse. Any health professional member of the general practice involved with the patient(s) could attend. The patient was rarely present as part of the program to facilitate scheduling, wider discussion and to allow greater numbers of patients to be discussed in the limited time available. Timing assumed 15 minute slots. A patient could be present at the insistence of the GP. GPs had access to billing for the Medicare Benefits Schedule chronic disease items for organising and coordinating a case conference. They are paid depending on the time spent on case conferencing (Supplement table 1). The diabetes case conferencing program remains ongoing in South Western Sydney. Time of the consultation was agreed mutually between the GP and endocrinologist, which could be at lunchtime (food was not provided) or a regular appointment time when there were no patients booked in for a consult. Endocrinologists work on certain days of the week; therefore, the appointments could only be booked on those days.

### Participants

General Practitioners were asked to prioritise and refer complex cases with longstanding management challenges where the HbA1c is ≥ 9% (75mmol/mol) and those with significant and frequent hypoglycaemia, with diabetes complications present (e.g. nephropathy, post hospital discharge, foot event in past 12 months), blood pressure over 160/100 mmHg, triglycerides 10+mmol/l and other patients that GPs would like to discuss. Each case conferencing consultation lasted approximately 15 minutes for each patient. Patient consent (verbal or written) prior to the consultation was required. During a case conferencing consultation, patient cases were reviewed (including e.g. medications, recent blood tests, diabetes duration, diabetes complications) and their multidisciplinary care needs were identified.

### Variables

Clinical variables included HbA1c, systolic blood pressure (SBP) and diastolic blood pressure (DBP), derived BMI, weight, blood lipids, serum creatinine, eGFR (calculated using CKI-EPI formula), urine albumin creatinine ratio, and their current medication from before, after and annually after the consultation where available. The Royal Australian College of General Practitioners (RACGP) treatment targets on the management of type 2 diabetes were used to describe the proportion of patients achieving clinical target/range before and after the consultation [17].

### Statistical Methods

Data were analysed using the Statistical Package for Social Sciences (SPSS) 27 software. Descriptive statistics included percentages for categorical variables, mean± standard deviation, or median (range) for continuous variables. Paired t-tests were used to compare pre- and post-consultation clinical data and repeated measures to explore changes in mean HbA1c, SBP, DBP, total cholesterol and weight over three years from year 2017–2020. To assess differential impact for those with more/less hyperglycaemia, the cohort was divided into quartiles based upon average HbA1c – (1) <7.5%, (2) 7.5–8.6%, (3) 8.7– 10.0%, (4) >10.1% with a paired t-test performed to compare the baseline and follow-up HbA1c within each group. Ages were grouped into quartile with approximately equal number of patients in each group: <55 years (group 1), 55–64 years (group 2), 65–74 years (group 3) and >75 years (group 4) and HbA1c was also compared within these groups. The Wilcoxon signed-rank test was used to compare the non-normally distributed pre- and post-case conferencing UACR and serum creatinine. McNemar test was used to see if there was difference in the paired group, to compare groups achieving clinical targets and if the groups were statistically significant before and after the intervention. All tests are two tailed with p <0.05 considered statistically significant. To investigate whether the intervention was more effective in reducing the HbA1c in any patient subgroups, a binary logistic regression using Bayesian method was performed and missing data values were imputed in multiple imputation by using fully conditional specification (FCS MI) method in SPSS. Two patient groups above and below the median difference in HbA1c were created. Patient groups included were gender (men and women), BMI groups according to RACGP classification criteria (18.5–24.9 kg/m^2^ = group 1, 25–29.9 kg/m^2^ = group 2 and ≥30 kg/m^2^ = group 3), diabetes duration (above and below median- 8 years), and age groups (above and below median age (64 years).

### Ethics approval

Ethics approval was granted by the South Western Sydney Local Health District Human Research Ethics Committee -2020/ETH00521.

## Results

**Figure 1:**
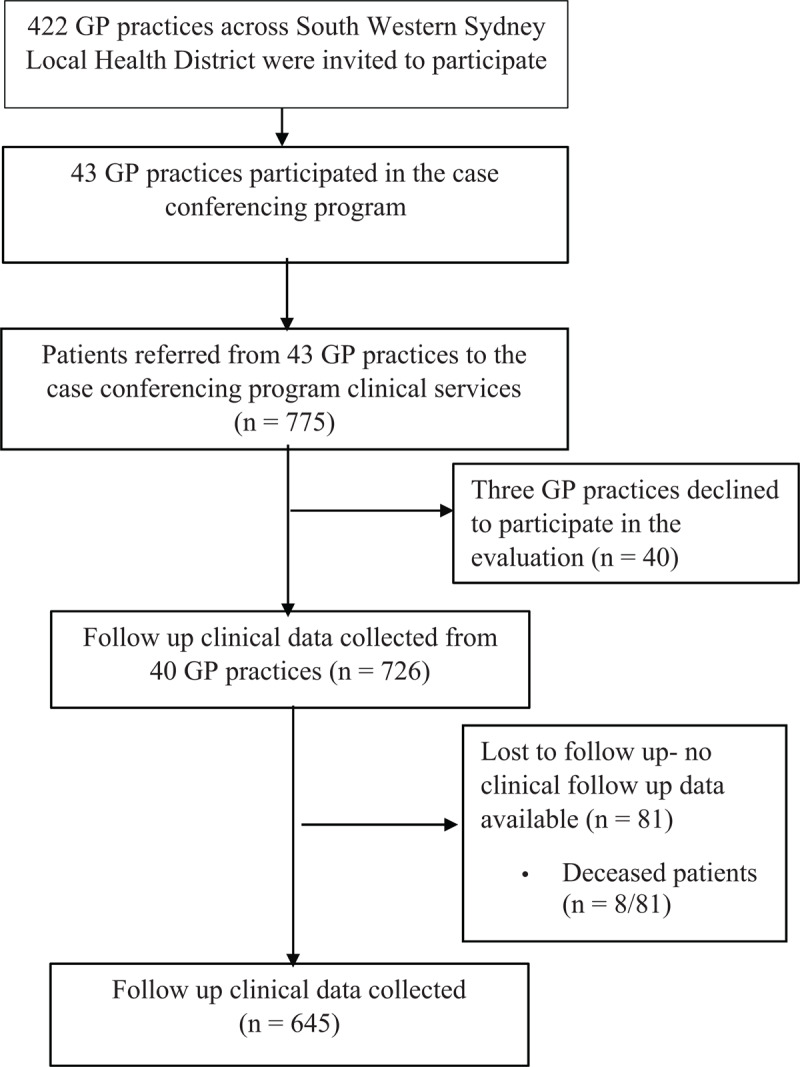
Flowchart of the clinical data collected from 43 general practices across South Western Sydney.

Data were available from 645/775 patients from 40/43 participating general practices (Figure 1). Of the 43 practices who participated and referred patients, only three refused to participate in the evaluation program. Of these, two practices were no longer participating and referred only 1–2 patients and the third practice had a change in ownership and GPs.

Of the 645 patients, 351 (54.4%) were men, mean age was 64 ± 15 years with median (range) diabetes duration eight (1–43) years, 96.4% had type 2 diabetes and 3.6% type 1 diabetes. Overall, 14.5% patients were smokers and 22.8% ex-smokers. Alcohol was consumed by 24.9% patients and 2.6% were ex-drinkers. Nineteen patients had more than one case conferencing consultation. The ethnicity of patients was not documented in most of the GP records and was therefore not included in the analysis. Forty-two patients were treated with insulin only (6.5%), 350 patients were treated with tablets/Glucagon-like peptide-1 receptor agonists (GLP1 RA) (54.3%), 203 (31.5%) patients received combined insulin/non-insulin therapy and 22 patients (3.5%) with diet alone. Diabetes complications were common with 26.7% patients having an eye complication, 22.2% kidney disease (14% had Chronic Kidney Disease with seven patients undergoing dialysis), 73.8% had cardiovascular disease (68.2% had hypertension), 9% had cerebrovascular disease, 3.7% had a lower limb amputation, 23.9% had depression and 49.9% patients had more than one complication. Mean HbA1c for patients (n = 56) lost to follow-up was 9.2% and 62.5% had cardiovascular disease. There were no baseline data for 25 patients.

The time between the baseline and follow up data collection was a median of 30 months (9–33 months). Table 1 shows change in clinical data between the initial case conferencing (baseline data) and the follow up data collection and Table 2 shows number of patients achieving clinical targets (%) before and after consultation.

**Table 1:** Change between baseline and follow-up clinical data collected. # Range was 0.1–585 mg/mmol. ## Range was 28–892 umol/L. Values are reported as mean ± standard deviation (except for UACR and serum creatinine), effect size is not reported for serum creatinine and UACR as a non-parametric test was performed.


CLINICAL VARIABLES	PATIENT (n)	BASELINE DATA	FOLLOW-UP DATA	p-VALUE	EFFECT SIZE

Weight (kg)	493	91.1 ± 23.6	87.9 ± 23.7	<0.001	0.32

Body Mass Index (kg/m^2^)	471	32.5 ± 7.4	31.2 ± 7.4	<0.001	0.36

Systolic Blood Pressure (mmHg)	591	134 ± 18	126 ± 12	<0.001	0.45

Diastolic Blood Pressure (mmHg)	591	77 ± 11	74 ± 12	<0.001	0.24

HbA1c (mmol/mol)	645	74 ± 22	63 ± 18	<0.001	0.59

HbA1c (%)	645	8.9 ± 2.0	7.9 ± 1.7	<0.001	0.59

Total Cholesterol (mmol/L)	591	4.6 ± 1.4	4.4 ± 1.9	0.007	0.11

Triglycerides (mmol/L)	588	2.3 ± 2.4	2.3 ± 4.0	0.83	0.01

High Density Lipoprotein (mmol/L)	567	1.2 ± 0.4	1.2 ± 0.3	0.15	0.06

Low-Density Lipoprotein (mmol/L)	548	2.4 ± 1.1	2.3 ± 1.0	<0.001	0.19

eGFR (Estimated glomerular filtration rate) mL/min/1.73m^2^	645	74.9 ± 22.4	73.8 ± 23.4	0.02	0.09

Urine albumin creatinine ratio (mg/mmol)# median (interquartile range)	439	2.2 (0.8–9.5)	2.5 (0.9–10.6)	0.97	

Serum creatinine## (umol/L) median (interquartile range)	643	80 (67–100)	80 (69–97)	0.09	


**Table 2:** Shows number of patients achieving clinical targets (%) before and after consultation.


CLINICAL VARIABLE	n	BEFORE CONSULTATION (% WITHIN TARGET/RANGE)	AFTER CONSULTATION (% WITHIN TARGET/RANGE)	p-VALUE FOR PRE/POST COMPARISON

HbA1c (%) (≤7%)	645	20.0	36.7	<0.001

Systolic blood pressure (≤130mmHg)	591	47	77	<0.001

Diastolic blood pressure (≤80mmHg)	591	71	81	<0.001

Total cholesterol (<4.0mmol/L)	591	36.4	42.8	0.003

Triglycerides (<2.0mmol/L)	588	56.6	66.5	<0.001

HDL (≥1.0mmol/L)	567	77.8	76.9	0.61

LDL (<2.0mmol/L)	548	36.1	41.2	0.01


There was a decrease in the prescription of non-insulin medication and insulin only, and an increase in a combination of insulin and non-insulin prescription. Table 3 shows medication changes occurred following the consultation and also shows the changes in the prescriptions of individual classes of diabetes medication before and after the consultation. There was an increase in insulin, DPP-4 inhibitors, GLP-1RA’s and SGLT2 inhibitors and a decrease in metformin prescription.

**Table 3:** Shows the changes in the prescriptions of individual classes of diabetes medication before and after the consultation in 645 patients.


MEDICATION	n	BEFORE (%)	AFTER (%)	p-VALUE

Non-insulin only	645	63.1	54.3	<0.001
	
Insulin only		8.5	6.5	0.06
	
Diet-controlled		3.9	3.4	0.58
	
Both non-insulin and insulin		21.9	31.5	<0.001
	
**MEDICATION CLASSIFICATION**		**BEFORE** (%)	**AFTER** (%)	**p** **-VALUE**
	
Biguanides		58.6	53.5	0.01
	
Sulphonylureas		27.4	27.9	0.84
	
DPP-4 inhibitors		21.1	24.3	0.08
	
SGLT2 inhibitors		16.3	22.9	<0.001
	
GLP-1 agonists		4.7	9.3	<0.001
	
Insulin		30.1	37.7	<0.001


Of the 90 patients who had CKD, 34.5% patients remained treated with metformin treatment after the consultation, 23.3% patients were no longer receiving metformin, and 8.9% patients were prescribed metformin post consultation and 33.3% patients were not on metformin treatment.

**Figure 2:**
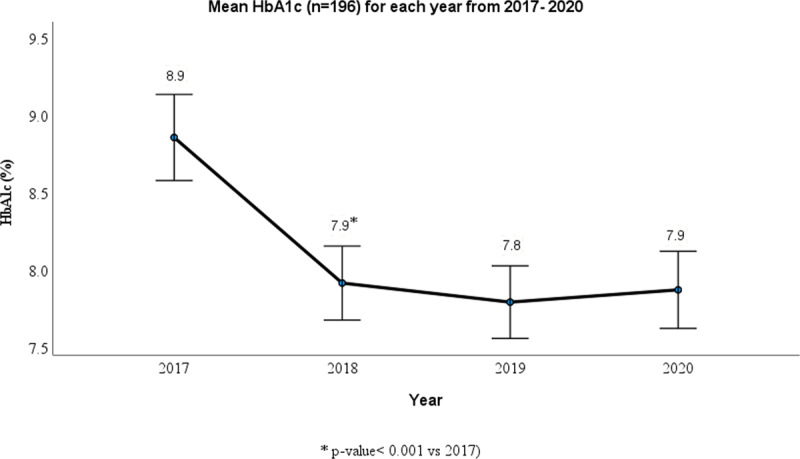
Shows the mean change in HbA1c for 196 patients each year from 2017–2020.

Among those with repeat data from 2017 (n=196), the HbA1c (Figure 2), SBP, DBP and weight (Supplementary figure 1) showed a significant reduction after one year and no subsequent reduction in the following 2 years. Table 4 shows that the HbA1c dropped significantly in the three highest quartiles.

**Table 4:** Shows change in mean HbA1c before and after consultation in four groups. Grouping was performed for gender (men and women) and HbA1c groups using quartiles and percentile for age groups.


GENDER	n	BASELINE HbA1c(%)	FOLLOW-UP HbA1c(%)	p-VALUE	EFFECT SIZE

Men	351	8.7 ± 2.0	7.8 ± 1.7	<0.001	0.58

Women	294	9.0 ± 1.9	8.0 ± 1.7	<0.001	0.62

**HbA1c GROUPS**	**n**	**BASELINE HbA1c**	**FOLLOW-UP HbA1c**	**p-VALUE**	

Group 1 = <7.5%	205	6.9 ± 0.8	6.4 ± 0.6	<0.001	0.58

Group 2 = 7.5–8.6%	192	8.6 ± 0.8	7.5 ± 0.7	<0.001	0.85

Group 3 = 8.7–10.0%	140	10 ± 1	8.6 ± 1	<0.001	0.76

Group 4 = >10.1%	108	11.7 ± 1.5	10.5 ± 1.8	<0.001	0.46

**AGE-GROUP (IN YEARS)**	**n**	**BASELINE HbA1c**(%)	**FOLLOW-UP HbA1c**(%)	**p-VALUE**	

18–54	163	9.6 ± 2.1	8.5 ± 2.1	<0.001	0.61

55–64	162	9.3 ± 2.0	8.1 ± 1.7	<0.001	0.65

65–74	165	8.8 ± 1.9	7.7 ± 1.4	<0.001	0.66

75–97	155	7.8 ± 1.4	7.2 ± 1.3	<0.001	0.50


The binary logistic regression showed no difference in effect among those above and below the median HbA1c by age, diabetes duration, gender and BMI groups according to RACGP classification criteria. However, HbA1c reduction was more likely to occur among those aged below 64 years (odds ratio (OR) = 1.91, 95% CI (1.25–2.93).

## Discussion

This is the first pre- post evaluation of patient-free diabetes case conferencing in a primary care setting. Despite only lasting approximately 15 minutes, this form of diabetes case conferencing was associated with significant reductions in glycaemia [mean HbA1c drop of 1% (11 mmol/mol), systolic blood pressure (mean drop of 8 mmHg), diastolic blood pressure (mean drop of 3 mmHg), lipids and weight. Glycaemic outcomes improved significantly in the first year in patients who visited their GPs annually for follow-up, and was then maintained in the following years. The proportion of patients achieving targets increased for HbA1c, blood pressure, total cholesterol, triglycerides and LDL after the consultation. There was also a significant improvement in HbA1c in all age groups and in both men and women. Patients who were hard to manage were referred by their GPs to an endocrinologist for case discussion. This study is built upon the vanguard diabetes case conferencing patient-free consultations in the South Western Sydney [18].

Besides the case conferencing sessions and associated travel, cancelled sessions and availability for sessions, the 0.7FTE endocrinologists were also involved in approaching new general practices to participate in the program and district wide primary care education activities. The degree of endocrinologist downtime remained dependent on the success of the local Primary Health Network general practice communication activities and subsequent number of general practices engaging in the case conferencing program. Initially before COVID-19, all case conferences were face to face. During the COVID-19 pandemic, case conferencing telehealth was used.

Recent evidence on diabetes care highlights that case conferencing programs can help close the gaps in service provision such as barriers between different settings, improve patient experience by accommodating more complex cases in primary care and reduces duplication of care [10,12]. In two smaller Australian case conferencing studies, patients were present during the consultations and reported statistically significant improvement in HbA1c, of 0.4% (4.7 mmol/mol) (n = 344) and 0.93% (10.2 mmol/mol) (n = 41) at six months and three years follow-up. Compared to these studies this study was different as the consultations were patient-free and included a larger number of cases discussed [10,12].

Several diabetes integrated care studies internationally have reported improvements in metabolic outcomes and cardiovascular risk factors, reduction in hospitalisation, improved quality of care, increased service cost-effectiveness, improved clinical outcomes among indigenous population and rural communities and an opportunity for shared learning [7,9,13]. Integrated diabetes care models such as case conferencing can potentially reduce and prevent hospitalisations in people with complex diabetes compared to usual care, further reducing the burden on health systems and reduction in diabetes related health costs [19]. One study (the Diabetes Integrated Care Initiative) from the UK implemented similar case conferencing consultations in East Cambridgeshire and Fenland where diabetes specialists provided support to primary care; however, no clinical evaluation was reported [3]. This study has reported only clinical outcomes.

In Australia, similar integrated diabetes care approaches (integrated general practice and specialist care) also reported a significant reduction in HbA1c from 0.4-1% (4.7 mmol/mol -10.2 mmol/mol), significant associated reductions in blood pressure and total cholesterol, improved patient and GP satisfaction and upskilling the general practitioners in their practice setting [8,10,11,12,18,20]. Randomised controlled trials investigating the effectiveness of similar models both virtually and face to face have reported clinically important improvements in glycaemic control, blood pressure and total cholesterol in the intervention arm [7,8,21]. Similar to previous case conferencing studies, this study has also reported statistically significant reductions in metabolic outcomes. A qualitative study will follow looking at patient and GP satisfaction, and areas of improvement for this case conferencing model.

According to the American Diabetes Association in their Standards of Medical Care, when possible, emphasis should also be placed on reducing cardiovascular risk [22]. Patients referred in this study were mostly at risk of developing complications, with 68.2% with hypertension (a major risk factor for CVD, microvascular complication and leading cause of kidney disease) [23] and some with existing chronic complications. The results from the United Kingdom Prospective Diabetes Study (UKPDS) study showed a relationship between the risk of cardiovascular complications and glycaemia and for every 1% decrease in HbA1c there was a 35% reduction in the risk of complications, 25% decrease in diabetes related death and 7% reduction in all-cause mortality over 10 years [24]. Additionally, results from the UKPDS observational study showed that each 10 mmHg decrease in systolic blood pressure was associated with reduction by 13% in microvascular complications and 11% in myocardial infarctions [25]. While the UKPDS risk engine [26] would help predict the benefits from combining these HbA1c, SBP, DBP, cholesterol and weight reductions over a 5 to 10 year horizon, our study included patients with a median duration of 8 years. Some of the benefits from early metabolic improvements might not accrue in these patients who already had their type 2 diabetes for many years (as shown by the continued reduction in eGFR in our study).

### Strengths and Limitations

Strengths of this study include that it was a district-wide program across an area with six public and three private hospitals including the largest number of patients participating. The study had a low loss to follow up rate of only 16.8% (n=130), 93% of the general practices involved in case conferencing participated and it is the first patient-free case conferencing. The study captured comprehensive data over a period of three years. This case conferencing model was shown to be suitable in both urban and rural settings where a number of participating practices support large multi-ethnic populations with varying socioeconomic status. Weaknesses include being a single arm intervention study without a control group. The lost to follow-up patients were those who changed their GPs in the middle of the treatment, receiving care from multiple practices and may have had different outcomes to those remaining with their GP. We were unable to capture the ethnicity for most patients; however the population ethnicity is diverse in all seven LGAs [16] and thus can likely be translated across similar geographical and population setting. We did not perform a sub-analysis by socioeconomic area of practice because according to census 2016, over 80% of the population in SWS reside in areas with Relative Socio-Economic Disadvantage (IRSD) Index decile below 5 [27]. Future studies will include a health economic analysis and qualitative investigation of the perspectives from the different participating healthcare professionals. For a 15-minute case conferencing discussion to have such a major effect lasting up to three years suggest this may be one of the more cost-effective interventions for people with diabetes, and a full health economic analysis is warranted.

## Conclusion

In summary, we have shown that this case conferencing model, where the patient is not present allows cases to be discussed quickly (which is not emphasised in the alternative patient present model) and is associated with substantial improvements in management of patients with complex type 2 diabetes. It can also help clinicians in identifying critical areas and reduce clinical inertia. The results from this study show that this model can make significant contribution to the diabetes management in the primary care setting compared to the existing fragmented approach with delayed or absent support. A randomised controlled trial, including a cost effectiveness/cost utility study would be ideal to test the robustness of these results.

## Additional Files

The additional files for this article can be found as follows:

10.5334/ijic.6545.s1 Supplementary Figure 1. It shows the change in mean SBP, DBP, total cholesterol and weight for each year from 2017–2020.

10.5334/ijic.6545.s2 Supplement Table 1. It shows the Medicare Benefits Schedule chronic disease items for organising and coordinating a case conference.
