# Supplementary material for: Effectiveness of Diabetes Case Conferencing Program on Diabetes Management
Source: Int J Integr Care. 2023 Jan 25;23(1):2. doi: 10.5334/ijic.6545 (PMC9881444; doi:10.5334/ijic.6545)

Supplementary figure 1 shows the change in mean SBP, DBP, total cholesterol and weight for each year from 2017- 2020

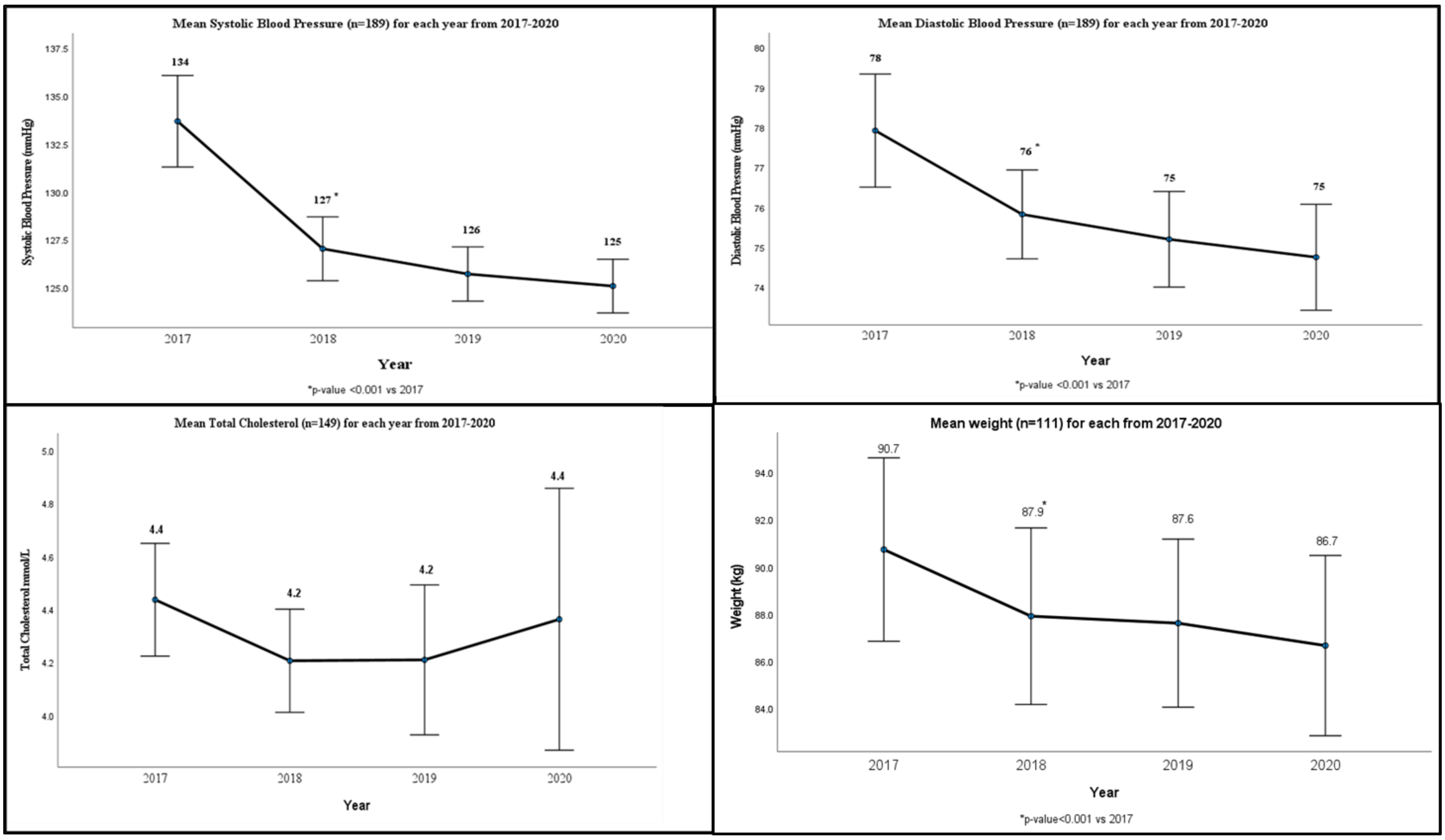

Supplement: Supplementary Figure 1. — It shows the change in mean SBP, DBP, total cholesterol and weight for each year from 2017–2020. [file ijic-23-1-6545-s1.pdf]
