# Supplementary material for: Effectiveness of Diabetes Case Conferencing Program on Diabetes Management
Source: Int J Integr Care. 2023 Jan 25;23(1):2. doi: 10.5334/ijic.6545 (PMC9881444; doi:10.5334/ijic.6545)
Supplement: Supplement Table 1. — It shows the Medicare Benefits Schedule chronic disease items for organising and coordinating a case conference. [file ijic-23-1-6545-s2.pdf]

Supplement table 1 shows the Medicare Benefits Schedule chronic disease items for organising and coordinating a case conference.

Items	Benefit	Time spent on case conferencing
735	$74.75	15-19 min
739	$127.85	20-39min
743	$213.15	40+mins
